# *Pasteurella multocida* Vaccine Candidates: A Systematic Review

**Published:** 2020

**Authors:** Saied Mostaan, Abbas Ghasemzadeh, Soroush Sardari, Mohammad Ali Shokrgozar, Gholamreza Nikbakht Brujeni, Mohsen Abolhassani, Parastoo Ehsani, Mohammad Reza Asadi Karam

**Affiliations:** 1. Department of Molecular Biology, Pasteur Institute of Iran, Tehran, Iran; 2. Drug Design and Bioinformatics Unit, Department of Medical Biotechnology, Biotechnology Research Center, Pasteur Institute of Iran, Tehran, Iran; 3. National Cell Bank of Iran, Pasteur Institute of Iran, Tehran, Iran; 4. Department of Microbiology and Immunology, Faculty of Veterinary Medicine, University of Tehran, Tehran Iran; 5. Hybridoma Lab, Department of Immunology, Pasteur Institute of Iran, Tehran, Iran

**Keywords:** *Pasteurella multocida*, Pasteurellosis, Vaccines

## Abstract

*Pasteurella multocida* (*P. multocida*) is the highly contagious causative agent of a broad range of diseases in animals as well as an occasional human pathogen. Economically significant infections caused by *P. multocida* include avian fowl cholera, rabbit snuffles, and hemorrhagic septicemia in cattle, goats and pigs. Chemotherapy of pasteurellosis infections has some limitations, such as high cost of treatment, low efficacy, and the possibility of therapy failure due to antibiotic resistance. Prophylactic immunization offers a safe and effective preventive measure in case of zoonotic diseases. Bacterins, live attenuated and some old traditional vaccines against pasteurellosis remain in use today, beside their limitations. However, the past few years have seen significant progress in research to identify modern, effective vaccine candidates, but there is no new vaccine produced by new strategies. While scientists should struggle with a lot of aspects to design vaccine producing strategies, this review shows how pasteurellosis vaccine evolved and the limitations in its application which need to be overcome.

## Introduction

*Pasteurella multocida (P. multocida)* is a nonmotile, gram negative, facultatively anaerobic, capsulated coccobacillus. It is routinely serotyped based on LPS and capsule, where the most pathogenic types are A1, A3, A4, B2, and D1. *P. multocida* colonizes at the nasopharynx, respiratory tract, gastrointestinal tract and other organs of many animals and induces a disease generally termed pasteurellosis. As with other opportunistic pathogens, under stress conditions the often commensal *P. multocida* becomes a pathogen, proliferating in the nasopharynx and spreading to lungs and other organs. It is responsible for avian fowl cholera, hemorrhagic septicemia in buffalo and cattle, enzootic pneumonia in cattle, lambs and goats, respiratory atrophic rhinitis of swine and snuffles in rabbits. As well as being a major cause of economic loss in production animals, *P. multocida* can also cause occasional, but severe, zoonotic infections in humans.

Antimicrobial agents provide one approach to control infections, but their shortcomings highlight the need to find other possible control measures such as prophylaxis to manage animal infections. Vaccination plays a vital role in improving the health and welfare of livestock and preventing animal to human transmission, thereby constituting a major public health strategy.

*P. multocida* infections in livestock industries can lead to high mortality rates and cause production losses, leading to considerable economic loss and hardship, especially in resource poor regions. This agent is responsible for 30% of total cattle deaths around the world and losses of one billion dollars annually in this industry in North America alone. In addition, it is responsible for considerable economic losses in pork and poultry industries worldwide. Humans are usually infected by contacting carrier or infected animals; disease can vary from soft tissue infection following animal bites to severe, fatal septicemia.

Pasteurellosis is a highly contagious disease affecting almost every animal species. Current control measures can be expensive and have limited efficacy. Therefore, vaccine design strategies leading to improved, cross-protective vaccines offer the best method of effective control. Since the researches to produce more potent, less impairing vaccines have been increasing recently, in this review, the focus was on the development of new vaccines and future perspectives to inform the scientists the way pasteurellosis vaccine has evolved and assist them in designing more targeted studies. Different key words for searching through the databases and data mining softwares such as Mendeley and EndNote were used to find the most relevant original articles.

### Whole cell vaccines

#### Killed vaccines:

These kinds of vaccine formulations, also called inactivated vaccines, bacterial lysates or bacterins, have utilized a range of preparation methods, such as killing of the bacteria by chemical agents, heat, drying, *etc*.

In one of the earliest studies, formalin killing of bacteria was applied resulting in a vaccine which was effective, but which elicited only homologous immunity. Further investigations with formalin killed vaccines by other scientists found levels of immunity ranging from 60% protection to no protection.

Samina *et al* evaluated a new route of injection for an oil-adjuvanted, killed *P. multocida* vaccine comprising serotypes A:1, A:2 and A:3, and concluded that injection in the wattle showed better or comparable protection indices (at 4 and 11 months of age, respectively) compared with the conventional subcutaneous route. In addition, the observation that no local reactions occurred following wattle vaccination is of economic importance, since subcutaneous vaccination may cause localized inflammatory reactions ^1^.

Arif *et al* studied immunopotentiation of an outer membrane protein preparation *via* anti-idiotype *P. multocida* vaccine versus bacterin vaccine in rabbits, and concluded that OMPs-anti-idiotype vaccine induced high levels of antibody titers, based on protection studies ^2^.

Waree *et al* assessed *P. multocida*-loaded microparticles as a hemorrhagic septicemia vaccine and concluded that alginate microparticles were safe and had the potential to induce protective immunity in mice even after storage for 6 months at either 4°*C* or room temperature ^3^.

Homayoon M *et al* used *P. multocida* serotype A inactivated with ferric chloride and adjuvanted with bacterial DNA (bDNA) and concluded that bDNA is effective as an immune adjuvant, and along with stimulatory bDNA represents promising new humoral and cellular immune enhancers for vaccination applications and also provides long-term protection against infection in mice ^4^.

A range of different adjuvants has been tested to increase the potency of killed vaccines. Results have been variable, with some studies showing no change, while others have achieved better potency and longer lasting immunity of up to 12 months. Outcomes also depended on the species and age of the vaccinated host, administration dose, and frequency of administration. Moreover, cross-protection against heterologous challenge was achieved in buffalo immunized with formalin-killed, oil-adjuvanted vaccine of *P. multocida* serotypes B:2 and B:5. Other attempts to stimulate heterologous immunity have included the use of multivalent vaccines containing up to 5 different serotypes. Results have shown a reduction of symptoms in calves, but not with fowl cholera. It is noteworthy to mention that vaccination with bacterins has multiple side effects, such as lack of cross serotype protection (heterologous protection), ineffective and short immunity, and the involvement of immunized animals in disease outbreaks, and lesions and inflammation at the site of injection.

Table 1 presents pasteurellosis vaccines and vaccine candidates.

**Table 1.** *P. multocida* vaccines and vaccine candidates

**No**	**Vaccine type**	**Animal**	**Experiment**	**Vaccine/Vaccine candidate**	**Challenge strain/serotype**	**Protection**		**Reference**

						**N**	**%**	
**1**	Killed	Turkeys	HI1	A:1, A:2, A:3	X-73	16/15	80/88	Samina, 1999
**2**	Killed	Rabbits	Challenge	B:2	B:2 serotype	8	100	Arif, 2013
**3**	Killed	Mice	Challenge	*P. multocida* loaded alginate MPs	B:2 serotype	10	6 log less than control group	Waree, 2013
**4**	Killed	Mice	Challenge/cytokine assay	*P. multocida* A strain PMSHI-9	*P. multocida* A strain PMSHI-9	17	100	Homayoon, 2018
**5**	Avirulent	Broiler chickens	Challenge	CU2 strain	P-1059	20	50	Bierer, 1969
**6**	Avirulent	Turkeys	Challenge	P-1059	P-1059	20	97.5	Bierer, 1972
**7**	Avirulent	Turkeys	Challenge	P-1059	X-73	30	90	Bierer, 1972
**8**	Avirulent	Turkeys	Challenge	CU strain	P-1059	10	100	Bierer, 1977
**9**	Avirulent	Turkeys	Challenge	CU strain M2283 P1580	P-1059	40	100	Dua, 1978
**10**	Avirulent	Broiler chickens	Challenge	CU strain	X-73	40	95–97.5	Rice, 1977
**11**	Avirulent	Turkeys	Challenge	Serotype 3	X-73	4	90–100	Singer, 1979
**12**	Avirulent	Holstein-Friesian calves	Challenge	A:3	A:3	11	NA	Chengapa, 1989
**13**	Avirulent	Holstein dairy calves	IgG IgM	Modified-live *P. multocida* vaccine	NA	179	5.3 fold more	Aubry, 2001
**14**	Avirulent	Swine	Cytokines assay	SPML3 vaccine	NA	10	NA	Zhang, 2007
**15**	Avirulent	Mice	Challenge	CNP-VRIL2S4 CNP-VRIL25	C44-8	15	90 60	Gao, 2007
**16**	Avirulent	Cattle	Challenge	A1	A6	6	5 log less than control group	Crouch, 2012
**17**	Avirulent	Mice	Challenge	N-PMT6	D	12	87.5	Kim, 2010
**18**	Avirulent	Pigs	IgG	N-PMT	D	3	Significantly increased	Kim, 2012
**19**	Avirulent	Ducks	Challenge	0818 strain fur mutant	0818 strain	65	62	Liu Q, 2019
**20**	Subunit	Piglet	Challenge	Truncated PMT	Type D	7	Significantly decreased in clinical symptoms	Riising, 2002
**21**	Subunit	Rabbits	IgG	PTE	D:3	15	Significantly increased	Suckow, 2008
**22**	Subunit	Pigs	Abs	rsPMT	Type D	9	Significantly increased	Chien, 2006
**23**	Recombinant	Mice	Abs	IL-6	C44-8	15	Significantly increased	Gao, 2009
**24**	Recombinant	Goats	Abs	Fimbrial protein	B: 2	15	Significantly increased	Mohd Yasin, 2011
**25**	Recombinant	Turkeys	Challenge	rFHAB2	x-73	40	75%	Tatum, 2012
**26**	DNA Vaccine	Mice	Challenge	tbpA	P-52	12	83.3	Singh, 2011
**27**	DNA Vaccine	Chicken	Challenge	pOMPHA	CVCC474	20	75	Gong, 2013
**28**	DNA Vaccine	Chicken	Challenge	ptfA g	CVCC474	25	100	Gong, 2018
**29**	Ghost	Mice	Challenge	NA	2365/A:7	10	100	Marchart, 2003
**30**	DNA Vaccine	Chicken	Challenge	NA	A:1	20	100	Herath, 2010

### Live attenuated vaccines

This kind of vaccine preparation strategy (live attenuated or avirulent) could be prepared based on multiple methods such as treating the bacteria in iron deficient environment, use of chemically mutagenic substances, and virulence genes deletion. Most of them have homologous protection but acapsular one showed to have heterologous protection partially. Protection range of this kind of formulations is up to 97.5% but route of infection, dose of administrations, quantity of bacteria in one dose, age of animal, type of animal, co-feeding by antibiotics may hinder the protection and its lasting. Some potent forms are *P. multocida* genes encoding toxins deletion, aroA deleted derivative of P. multocida, marker-free aroA derivative of *P. multocida*, live temperature-sensitive *P. multocida* mutant and *P. multocida* cexA mutant (PBA875). Acapsular *P. multocida* strain (AL18) and *P. multocida* mutant show that candidate vaccine protein has some advantages like induction of heterologous protection, cellular immunity induction, longer lasting protection, but they may have some serious disadvantages like causing systemic infection, disease outbreaks, no or weak protection against fowl cholera in chickens, and weight gain.

Bierer *et al* in 1969 compared attenuated live *P. multocida* vaccine given in the drinking water every two weeks to an injected oil-based bacterin administered to turkeys and concluded that live water vaccine given every 2 weeks is superior (p<0.05) to oil-based bacterin injected once or twice, and that the oil-based bacterin was better than no treatment at all ^5^.

Bierer *et al* in 1972 evaluated immunologic response of turkeys to an a virulent *P. multocida* vaccine in the drinking water and concluded that an avirulent vaccine used 4 weeks after the use of an oil-based bacterin was effective in reducing the total number of infected turkeys at 9, at 16, and at 30 weeks post-vaccination, but that at 30 weeks post-vaccination, the reduction of the total number infected was not as great and the duration of the immunity to the virulent homologous (P-10S9) strain appeared to be similar to the duration of the immunity to each of the two virulent heterologous (P-1662 and X-73) strains 6.

Bierer *et al* in 1972 also evaluated immunologic response of turkey poults of various ages to an avirulent *P. multocida* vaccine in the drinking water and concluded that turkey poults vaccinated on 35 days of age and poults vaccinated on 20 days of age experienced an excellent immunological response by 5 weeks of age. This response was less evident but, for the most part, it was still present at 12 weeks of age ^6^.

Bierer in 1977 evaluated the effect of various concentrations of the Clemson University (CU) *P. multocida* vaccine on the immune response against fowl cholera disease in turkeys and showed its dose dependency on oral use where high doses had near to full protection ^7^. This data was reconfirmed by Coates *et al* in 1977 by designing different experiments ^8^.

Dua *et al* evaluated local humoral immunity induced by a live avirulent fowl cholera vaccine and concluded that CU strain of *P. multocida* appeared to generate a local humoral immunity in the respiratory system whereas the bacterin did not ^9^.

Rice *et al* evaluated vaccination routes of chickens with a live, avirulent *P. multocida* vaccine and concluded that the subcutaneous route produced the greatest degree of protection in all experiments and protection levels were as high as 95 and 97.5% in broilers vaccinated subcutaneously and no undesirable lesions or cheesy masses were formed under the skin in the back of the necks of broilers 7.

Singer *et al* evaluated avirulent live *P. multocida* vaccine for drinking water and aerosol administration against turkey cholera and concluded that satisfactory degree of protection and even cross-immunity is evident from challenge experiments with both homologous and heterologous serotypes given as a spray ^10^.

Catt DM *et al* studied the efficacy of live *P. multocida* vaccine for the prevention of experimentally induced bovine pneumonic pasteurellosis and concluded that live pasteurella vaccine is effective against experimental *P. multocida* infection in calves ^11^.

Prantner *et al* studied comparison of two vaccine strains and a field isolate and concluded that in addition to bacteremia and mortality, the ability to replicate extracellular and to produce septicemic lesions may be associated with strain virulence. Therefore, the bacterial components expressed by *P. multocida* that resist the bactericidal activity of serum and phagocytes may be important virulence determinants for *P. multocida* serotype A:3,4 ^12^.

Aubry *et al* studied health and performance of young dairy calves vaccinated with a modified-live *Mannheimia haemolytica (M. haemolytica)* and *P. multocida* vaccine and concluded that *M. haemolytica* and *P. multocida* vaccine, given twice 2 weeks apart, was effective in increasing titers of antibodies against *M. haemolytica* in young dairy calves but did not improve calf performance or health ^13^.

Zhang *et al* studied unmethylated CpG oligodeoxy-nucleotides (CpG ODN) and immune responses of pig-lets containing CpG ODN and swine *P. multocida* living vaccine and came to conclusion that the therapeutic uses envisioned for these ODNs (as vaccine adjuvants and immunoprotective agents) may be applicable in animal husbandry due to their ability to modulate the immune response towards a Th1-like response ^14^.

Gao *et al* studied shuffling of pig *interleukin-2* gene and its immunity enhancement in mice to *P. multocida* vaccine and indicated that shuffled IL-2 cloned into VR1020 eukaryotic plasmid (VRIL2S) entrapped with chitosan nanoparticles is a novel safe and effective adjuvant to boost the specific immunity and resistance of animal against infectious pathogen, which could facilitate the development of highly promising powerful adjuvant ^15^.

Crouch *et al* studied cross protection of a *M. haemolytica* A1 Lkt-/*P. multocida*_hyaE bovine respiratory disease vaccine against experimental challenge with *M. haemolytica* A6 in calves and concluded that the formulation can protect calves against clinical disease following challenge ^16^.

Kim *et al* studied vaccine potential of an attenuated *P. multocida* that expresses only the N-terminal truncated fragment of *P. multocida* toxin in pigs and concluded that pigs vaccinated with the mutant showed significantly higher rates of antibody induction and lower nasal conchal (turbinate) scores for atrophic rhinitis than controls, which suggests that this mutant strain may be a good candidate for a live attenuated vaccine ^17^.

Harper M *et al* reviewed the myriad properties of *P. multocida* lipopolysaccharide and concluded that live attenuated vaccines gave broad protection, and their efficacy is independent of LPS structure ^18^.

Oslan SNH *et al* also studied stability of live attenuated vaccine gdhA derivative *P. multocida* B:2 by freeze drying method as an animal vaccine and proved its stability ^19^.

Liu Q *et al* studied identification of fur in *P. multocida* and the potential of its mutant as an attenuated live vaccine and concluded that ducks that were orally inoculated with the fur mutant strain demonstrated 62% protection efficacy against severe lethal challenge with the wildtype *P. multocida* ^20^.

### Acellular vaccines

#### Subunit vaccines:

This kind of vaccines called “second generation vaccines” which are individual or fusion immunogenic part(s) of the pathogens like proteins (polysaccharides). Capsular antigen of pasteurella was first used as the subunit vaccine against pasteurellosis. The Lipopolysaccharide (LPS) of *P. multocida*, *P. multocida* Toxin (PMT), Dermonecrotic Toxin (DNT), bacterin-toxoid (BT), native and iron regulated or heat modified Outer Membrane Proteins (OMPs), bacterial adhesins, purified siderophore receptor proteins lipoprotein E (PlpE) and many others are studied so far.

Suckow *et al* evaluated immunization of rabbits against *P. multocida* using a commercial swine vaccine including Inactivated *P. multocida* Toxin (IPMT) and concluded that a commercial swine vaccine stimulates antibody activity to and protective immunity against *P. multocida* heat labile toxin in rabbits ^21^.

Riising *et al* studied protection of piglets against atrophic rhinitis by vaccinating the sow with a vaccine against *P. multocida* and concluded that the vaccine conferred very effective protection against atrophic rhinitis, because the incidence of clinical signs and the level of atrophy of the conchae were much lower among the pigs from the vaccinated sows than among the pigs from the control sows ^22^.

Suckow *et al* used Potassium Thiocyanate Extract (PTE) produced from *P. multocida* to vaccinate pasteurella free rabbits and concluded that PTE can be used to stimulate protective immunity to a heterologous strain of *P. multocida*, with stronger immunity generated by subcutaneous than intranasal vaccination ^23^.

This kind of preparation has its noteworthy advantages like having no live components, so the risk of inducing the disease is low, and the vaccine is safer and more stable than live attenuated one and can be administered to newborn and people suffering from weakened immunity.

Some weaknesses of this technique can be enumerated as well. Isolated proteins, if denatured, may bind to different antibodies than the protein of the pathogen. It is important to determine which combination of antigenic properties will produce an effective immune response with the correct pathway; a response may be elicited, but with no guarantee of memory for future responses and several doses must be given for proper life-long immunity.

### Recombinant vaccines

This kind of vaccine formulation called third generation vaccines (or part of second generation) is evolutionary generation and plays a main role in combating infectious diseases and also has overlap with subunit vaccines. In case of *P. multocida*, the first attempt was non-toxic recombinant derivative of the *P. multocida* toxin (rPMT).

Liao CM *et al* studied immunogenicity and efficacy of three recombinant subunit *P. multocida* toxin vaccines against Progressive Atrophic Rhinitis (PAR) in pigs and concluded that non-toxic rsPMT (Short fragments of recombinant subunit *P. multocida* toxin) proteins are attractive candidates for development of a subunit vaccine against PAR in pigs ^24^. This group later used a vaccine combining 3 different short recombinant proteins and came to conclusion that vaccine could be used to provide protective immunity for controlling the prevalence and severity of PAR among farm-raised swine ^25^.

Al-Hasani K *et al* in 2007 studied 129 proteins as secreted, located in the outer membrane, or lipoproteins and identified 12 immunogens of *P. multocida* among which 6 were novel potent immunogens in chickens ^26^.

Other recombinant proteins are P6-like protein, OmpH, OmpA, adhesive protein (rCp39), filamentous hemagglutinin peptides (rFHAB2), *P. multocida* lipoprotein E (PlpE), OmpH and lipoprotein E (PlpE) genes fusion (PlpEC-OmpH), recombinant clone ABA-392, and sub-clone CSI57 J.

Gao *et al* studied promotion of immunity of mice to *P. multocida* and hog cholera vaccine by pig *interleukin-6* gene and CpG motifs and concluded that VR1020 plasmid containing pig *interleukin-6* gene on chitosan nanoparticles (VPIL6C) could better promote the immunity and resistance of mice against pasteurellosis than conventional bivalent vaccine and facilitate the development of effective adjuvant to enhance the immunity of animal against infection ^27^.

Also, it is noteworthy to mention that soluble and insoluble PlpE both in mice and chickens protected against heterologous challenges ^28^. Peptides of OmpH protected mice against homologous, and chickens against heterologous challenges ^25^. Lastly, fragments of recombinant filamentous haemagglutinin protein Fha-B2 elicited protection in turkeys ^29^.

Mohd Yasin IS *et al* evaluated efficacy of an inactivated recombinant vaccine encoding a fimbrial protein of *P. multocida* B:2 against hemorrhagic septicemia in goats and concluded that inactivated recombinant vaccine significantly provides significant protection against high dose challenge and enhances the stimulation of the local and systemic immunities ^30^.

Tatum *et al* studied cross protection against fowl cholera with the use of recombinant *P. multocida* FHAB2 peptides vaccine and concluded that vaccination with rFHAB2 (recombinant Filamentous Hemagglutinin) peptides significantly protected turkeys against lethal challenge from both *P. multocida* serotypes ^31^.

Same advantages and disadvantages of subunit vaccine could be indicated for this type of preparation and also protein folding and modifications are extra disadvantage.

### DNA vaccines

This kind of vaccines called third or fourth generation vaccines. DNA vaccine derived from *P. multocida Toxin (PMT)* gene was first used in this field.

Singh S *et al* studied immune response to DNA vaccine expressing transferrin binding protein, a gene of *P. multocida* and concluded that the bicistronic DNA vaccine provided superior immune response and protection level following challenge as compared to monocistronic construct ^32^.

Gong *et al* studied immune responses and protective efficacy of a novel DNA vaccine encoding outer membrane protein (OmpA and OmpH and fusion) of avian *P. multocida* and concluded that protection provided by divalent combination and fusion DNA vaccines was superior to that provided by monovalent DNA vaccines and the protective efficacy in chickens immunized three times with the fusion DNA vaccine was equivalent to the protective efficacy in chickens vaccinated once with the attenuated live vaccine ^33^.

Gong *et al* in 2018 studied the ptfA chitosan nanoparticle DNA vaccine against *P. multocida* and its immune response in chickens and concluded that chitosan was able to enhance the immune response to a naked DNA vaccine based on the *ptfA* gene of *P. multocida* ^34^.

Outer membrane protein DNA vaccines such as OMP-DNA vaccines (pOmpH, pOmpA, pOmpHA), OmpH, PlpEN and PlpEC, divalent combination of pc-DNA-OmpH +pcDNA-OmpA, pOmpH +pOmpA, pc-DNA-OmpH, pOmpH and pcDNA-OmpA, pOmpA and some others are the result of scientist attempts to achieve potent DNA vaccine against *P. multocida*.

There are some advantages and disadvantages for this kind of vaccine preparation strategy like having no risk for infection, antigen presentation by both MHC class I and class II molecules, polarise T-cell response toward type 1 or type 2, immune response focused on antigen of interest, ease of development and production, stability for storage and shipping, cost-effectiveness, not requiring peptide synthesis, expression and purification of recombinant proteins and use of toxic adjuvants, long-term persistence of immunogen, *in vivo* expression ensuring protein resemblance to normal eukaryotic structure, with accompanying post-translational modifications, being limited to protein immunogens (not useful for non-protein based antigens such as bacterial polysaccharides), risk of affecting genes controlling cell growth, possibility of inducing antibody production against DNA, possibility of tolerance to the antigen (protein) produced, potential for atypical processing of bacterial and parasite proteins.

### Other vaccine strategies

Marchart J *et al* studied *P. multocida* ghosts as new vaccine candidates and showed animals which received 1.15×10^8^ ghosts and a challenge dose of up to 60 *cfu* manifesting 100% protection. According to these results, they suggest ghosts of *P. multocida* as new vaccine candidates ^34^.

Herath *et al* studied iron-inactivated *P. multocida* A: 1 vaccine adjuvanted with bacterial DNA and suggested that use of bacterial DNA as an adjuvant can improve the cost-effectiveness of inactivated veterinary vaccines for their use in developing countries ^35^.

Ren W *et al* studied dietary L-proline supplementation immunostimulatory effects on mice immunized by inactivated *P. multocida* vaccine and came to conclusion that dietary proline supplementation confers beneficial immunostimulatory effects ^36^. There is no evidence for Killed But Metabolically Active (KBMA) vaccine strategy against *P. multocida* yet.

## Conclusion

In case of pasteurellosis vaccine potency evaluation, high serum levels of IgG antibodies do not mean clearance of or resistance to pasteurella infection but rather is indicative of chronic infection, so vaccine or vaccine candidate potency studies based on elevated IgG levels, seem not to be a concern.

Several pasteurella outer membrane proteins have potential targets for vaccine development, *e.g*., antisera against the outer membrane protein Oma87 protected mice against a lethal dose challenge of *P. multocida*. Vaccination with *P. multocida* strain P-1059, recombinant adhesion protein Cp39, protected chickens from challenge with strain P-1059 (Serotype A:3) and strain X-73 (Serotype A:1). OmpH-specific antibodies were more effective than OmpA-specific antibodies in controlling *P. multocida* growth in mice, presumably by enhancing PMN phagocytosis. Full-length OmpH was more effective than shorter fragments of it as a vaccine against *P. multocida* isolated from a case of atrophic rhinitis in a mice challenge model. OmpA elicits a strong antibody response, but it has no protective potential in a mice model of infection. Type 4 fimbria subunit of serotype A, B, and D strains has good potential as a vaccine candidate; but, potent vaccine was only reported for the fimbria protein from serotype B:2 against hemorrhagic septicemia in goats.

Using *in silico* tools, 98 avian strain *Pm70* genes and 107 non toxicogenic porcine strain 3480 genes were selected as encoding putative OMPs. Out of them, 71 recombinant proteins, mostly insoluble, were cloned, expressed and purified and tested for their immunity improvement potential. Only one of them, pasteurella lipoprotein E (PlpE), was found as a good vaccine candidate against *P. multocida* in chickens and mice challenges, which confirmed previously reported results using the PlpE cloned from the avian serotype A:1 strain X-73. This fact that PlpE knockout mutant strain retained full virulence shows it has no role in virulence of *P. multocida*. Conjugated vaccines including multiple antigens, such as OmpH plus PlpE peptides, have also shown promise. There are some other vaccine candidates such as filamentous hemagglutinin protein (FhaB2), iron-regulated Omps, LPS, *etc*. and vaccination with peptides derived from FhaB2 protected turkeys from fowl cholera upon challenge with *P. multocida* P1059. LPS seems to be a major virulence factor and immunogen of *P. multocida*, but its use as a vaccine candidate is influenced by structural heterogeneity of different serovars. Several *in vivo*-expressed surface antigens have been identified as potential vaccine candidates.

Some Omps taking role as iron-regulators which are expressed during *P. multocida* infection have been studied so far as potential immunogens. For instance, the 96 *kDa* heme acquisition system receptor (HasR) protein is a conserved Omp which is exposed in surface of most of the *P. multocida* isolates. HasR is expressed when iron is low to be acquired *in vivo* and induces protection against bovine *P. multocida*. Serotype A:1 strain of *P. multocida* whole-cell vaccine has also been explored based on inactivation by treatment with high iron concentrations.

A few bacterin and/or toxin based vaccines are available commercially. A commercial vaccine against fowl cholera (chickens and turkeys), sold as CholeramuneM, Multimunem, or M-Ninevax-C, is available and is based on a freeze-dried preparation of a live, avirulent avian isolate of *P. multocida* M-9 strain (A serotype A:3-A:4 cross). A trivalent combination vaccine against fowl cholera (for ducks, chickens, and other poultry) and rabbit pasteurellosis, sold as Landavax, is available as an inactivated bacterin oil emulsion of *P. multocida* serotype A:1, A:3, and A:4 strains.

Recent studies in genetic, biochemical, and virulence factors of *P. multocida* and other Pasteurellaceae family members resulted in remarkable understanding of disease mechanisms of these organisms and led to the development of new non-bacterin vaccines, several of which are now available commercially for animal use.

Our interactions with pets and other domestic and wild animals are unlikely to diminish in the future. Mounting evidence suggests that such contacts that result in *P. multocida* infection can lead to outcomes ranging from benign to disastrous. Considering the high prevalence of pasteurella species as part of the microbiota of domestic and wild animals, it is suggested to consider zoonotic transmission of *P. multocida* as a serious risk for infection.
